# Spirulina supplementation effects on small ruminants performance and product attributes: a meta-analysis

**DOI:** 10.5713/ab.24.0835

**Published:** 2025-04-04

**Authors:** Frediansyah Firdaus, Tanda Sahat Panjaitan, Malik Makmur, Bayu Andri Atmoko, Yudi Adinata, Noor Hudhia Krishna, Retno Widiyawati

**Affiliations:** 1 Research Centre for Animal Husbandry, National Research and Innovation Agency, Cibinong Science Centre, Bogor, Indonesia; 2 Animal Feed and Nutrition Modelling (AFENUE) Research Group, IPB University, Bogor, Indonesia

**Keywords:** Antioxidant, Growth Performance, Meat, Meta-analysis, Small Ruminant, Spirulina

## Abstract

**Objective:**

The research aims to conduct a meta-analysis evaluating the nutritional benefits of Spirulina supplementation on growth performance, rumen function, antioxidant status, blood biochemistry, fatty acid profile, meat quality, and eating quality in small ruminant, specifically goats and sheep.

**Methods:**

A systematic review and meta-analysis were conducted to determine the impact of Spirulina supplementation on various aspects of small ruminant performance and product attributes. Electronic databases (Scopus, PubMed, and Google Scholar) were searched for relevant studies published between 2012 and 2023. From the 350 identified studies, 24 matched the inclusion criteria of original research. A meta-analysis was performed using OpenMEE software. Publication bias was assessed using the precision-effect test (PET)-precision-effect estimate with standard errors method and Rosenthal’s fail-safe N analysis, visualised through PET regression plots in JASP software. Standardised mean differences between Spirulina-supplemented and control groups were calculated to determine the effect magnitude.

**Results:**

Meta-analysis show that Spirulina supplementation significantly enhances growth performance, meat quality, and antioxidant status in small ruminants compared to controls. Supplementation increases average daily gain and reduces feed conversion ratio. Additionally, Spirulina supplementation elevates antioxidant enzyme activities, including catalase, glutathione peroxidase, glutathione, and superoxide dismutase. Meat quality parameters, such as fat melting point, intramuscular fat percentage, and pH, are also positively affected. Blood biochemistry markers, including alanine transaminase, aspartate transaminase, cholesterol, haemoglobin, low-density lipoprotein, total protein, urea, and white blood cell counts, remain within normal physiological ranges. Moreover, Spirulina supplementation favorably alters fatty acid composition, increasing the proportion of eicosapentaenoic acid and decreasing the proportion of palmitic acid. Supplementation duration ranged from 14 to 182 days, with doses between 0.014 to 2.14 g/kg body weight/day.

**Conclusion:**

Spirulina supplementation has potential effects on small ruminant productivity, especially growth performance, health and meat quality. Spirulina can be a valuable dietary addition for small ruminant. Further research is warranted to optimise supplementation strategies and understand underlying mechanisms.

## INTRODUCTION

The global population is rapidly expanding, driving a surge in demand for animal protein. Meat consumption has increased significantly, fuelled by rising living standards and urbanisation [1,2]. As a critical source of essential nutrients (such as protein, zinc, iron, selenium, calcium, and vitamin B12), meat plays a vital role in human diets. Small ruminants, particularly sheep and goats, offer a promising option to meet this growing demand. With a global population of 2.18 billion head, representing 56% of the world’s ruminant population, these animals contribute substantially to meat production [3]. Their adaptability to diverse environments and efficient feed conversion makes them valuable assets for many farming systems, especially in the face of climate change. Developing small ruminant livestock to produce healthy, sustainable, and high-nutritional-value meat is essential.

Recent research indicates that incorporating phytonutrients into livestock diets can improve animal health, resulting in improved meat quality and increased nutritional value for consumers [4]. Identifying novel feed sources with high nutritional efficiency is crucial for sustainable livestock production. Spirulina (*Arthrospira sp*.) is filamentous cyanobacteria widely used as a dietary supplement [5]. Spirulina offers potential benefits for small ruminants by improving their growth, health, and product quality [6]. Spirulina’s mechanism of action involves activating cellular antioxidant enzymes, inhibiting lipid peroxidation, and counteracting free radicals, thereby enhancing overall health and vitality of the animals [7]. Importantly, Spirulina increase feed intake in small ruminants fed low-quality diets, consequently boosting their protein intake and overall feed consumption [8–10].

Numerous studies have investigated the effects of Spirulina supplementation on small ruminant performance, reporting positive impacts on growth performance, rumen function, fatty acid profiles, antioxidant status, and meat quality [11–15]. However, inconsistencies in study designs, sample sizes, and outcomes hinder definitive conclusions about the overall impact of Spirulina supplementation. A meta-analysis is essential to synthesise the available evidence and identify consistent patterns in the effects of Spirulina on small ruminant production. By combining and analysing multiple studies [16–18], a meta-analysis can provide a more robust understanding of Spirulina’s benefits and inform evidence-based recommendations for its use in small ruminant farming.

It is hypothesised that Spirulina supplementation enhances overall small ruminant performance. This study aims to evaluate the impact of Spirulina supplementation on growth, rumen function, fatty acid profiles, antioxidant status, meat quality, and eating quality. The findings will contribute to understanding Spirulina’s potential as a sustainable and effective feed supplement for small ruminant production.

## MATERIALS AND METHODS

### Paper searching

**Figure 1:**
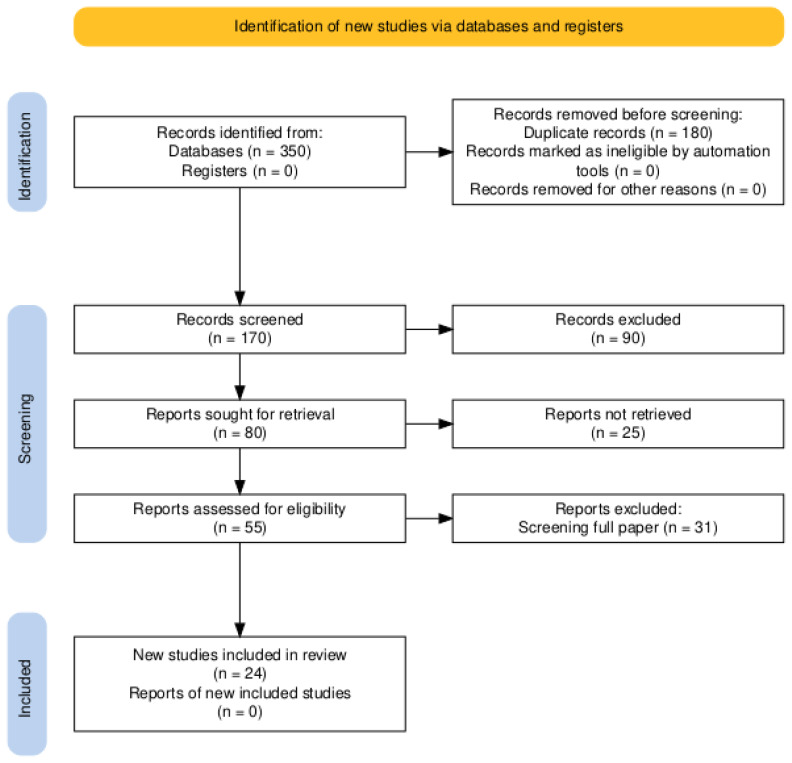
A PRISMA flow diagram visually represents the literature search and selection process employed in this meta-analysis. Initially, 350 papers were identified as potentially relevant to Spirulina. Adapted from Haddaway et al with CC-BY [19].

A systematic and comprehensive literature review was conducted between January and May 2024, following the Preferred Reporting Items for Systematic Reviews and Meta-Analyses guidelines [19], with paper numbers outlined in Figure 1. Published papers were retrieved from Scopus, PubMed, and Google Scholar databases. Keyword searches were conducted using the Population, Intervention, Comparison, and Outcome framework [20], with the following terms: population (goat and sheep), intervention (Spirulina), comparison (control), and outcomes (growth performance, rumen function, antioxidant status, blood biochemistry, fatty acid profile, meat quality, and eating quality). Recent studies conducted over the past 10 years, published between 2012 and 2023, were included. Inclusion criteria, adapted from Trevi et al [21], were: 1) original research on sheep or goats, 2) published in English, 3) Spirulina as a feed supplement, and 4) reporting mean values, standard deviation or standard error, and sample size for relevant variables.

### Data extraction

Data for meta-analysis were extracted from selected papers (Table 1) and then compiled and analysed. The following data were collected: (1) First author and publication year; (2) Mean value of the treatment group and control groups; (3) standard deviation of the mean values for treatment groups and control groups; (4) number of livestock in the treatment group and control groups (Nc); (5) livestock species (sheep, goat); (6) Spirulina usage dosage; and (7) study location.

### Analysis of data

A meta-analysis was conducted using Open Meta-Analyst for Ecology and Evolution software, a statistical tool designed for analysing ecological and evolutionary data [22]. The effect sizes were calculated as standardised mean differences using Hedges’ g, comparing Spirulina-supplemented diets to control diets. Cohen’s d effect size interpretation guidelines were used to interpret the magnitude of the overall effect: 0.2 representing a small effect, 0.5 a medium effect, and 0.8 a large effect [23]. After data inspection, a random effects model was employed to determine the overall effect, as a fixed effects model was not deemed appropriate.

The applied mathematical models for data analysis are as follows:


(1) $$Y_{i}=\mu +\tau_{i}+{ɛ}_{i}$$

(2) $$Q=\sum \left(w_{i}ES_{i}^{2}\right)-\frac{{\left(\sum \left(w_{i}ES_{i}^{2}\right)\right)}^{2}}{\sum w_{i}}$$

(3) $$\tau^{2}=\frac{Q-df}{C}$$

(4) $$I^{2}=\frac{\left(Q-(k-1)\right)}{Q}\times 100$$

where yi is the diversity of effect size, μ is the mean true effect, τi is the diversity of true effect size and ɛi is error sampling [16]. There are three measures used to measure the level of heterogeneity between studies, namely Cochran’s Q, index I^2^ (with thresholds of <25%, 25% to 75%, and >75% for low, moderate, and high heterogeneity, respectively), and tau-squared (τ^2^), calculated using the DerSimonian and Laird method. Where wi is the weight of each study, ES is the effect size value of each study, k is the number of studies analysed, df is the degrees of freedom and C is the overall estimation value. Publication bias was examined for the 4 items with the largest sample sizes using the precision-effect test-precision-effect estimate with standard error squared (PET-PEESE) method [24] and Rosenthal fail-safe N analysis [25], and visualised trough PET regression plots in Just Another Statistics Package (JASP) software (version 0.18.3).

## RESULTS AND DISCUSSION

### Study characteristic

A flowchart illustrating the study selection process is presented in Figure 1. An initial search of electronic databases (Scopus, PubMed, and Google Scholar) yielded 350 potential articles. After removing duplicates, 170 unique articles were screened, resulting in 80 relevant studies. Subsequent abstract screening based on data availability, sample size, and variable alignment eliminated 25 articles, leaving 55 for full-text review. Further screening for standard deviation or standard error of each independent variable resulted in the exclusion of 31 articles. Ultimately, 24 studies meeting inclusion criteria were included in the meta-analysis (Table 1 [12,14,15,26–45]).

The included studies, published between 2012 and 2023, originated from nine countries: Russia, Oman, Australia, Bangladesh, China, Kuwait, Egypt, the USA, and Indonesia. Most studies reported Spirulina supplementation duration ranging from 14 to 182 days, with dosages varying from 0.014 to 2.14 g/kg body weight (BW)/day. Administration methods included feed mixing, drinking water and drench peroral supplementation, as reported by Mansour and Zeitoun [13], Malau-Aduli and Holman [28], Holman et al [29], Malau-Aduli and Kashani [32], Kashani et al [36], Holman et al [42], and Flakemore [44].

### Chemical composition and nutritive value of Spirulina

Spirulina (*Arthrospira sp*.) a filamentous cyanobacterium with a spiral shape, is a rich source of nutrients [5]. As noted by Jung et al [46], Spirulina is remarkably adaptable and can thrive in harsh conditions, including saline environments. Renowned for its high protein content, Spirulina provides a complete amino acid profile and is abundant in vitamins and minerals (Table 2 [12,27,28,31,42,47–51]). Its protein content is notably twice that of soybeans, and it is a significant source of essential polyunsaturated fatty acids, such as omega-6 [52].

### The benefits Spirulina on growth and body conformation

The benefits of Spirulina supplementation on growth performance and body conformation of small ruminants are presented in Table 3. The table includes the cumulative effect size and 95% confidence interval (CI) for various levels of Spirulina supplementation. Meta-analysis results indicate that Spirulina supplementation significantly improved the growth performance of small ruminants. Specifically, average daily gain (ADG) increased significantly (d = 1.2±0.4, large effect size) compared to control groups. Conversely, feed conversion ratio (FCR) decreased significantly (d = −1.64±0.42, large effect size) after Spirulina supplementation.

While a few studies reported non-significant increases in body weight gain with Spirulina supplementation, most studies found significant improvements in body weight gain of small ruminants. For example, El-Sabagh et al [40] reported that lambs fed a formulated basal diet to meet nutrient requirements according to NRC [53] and supplemented with Spirulina platensis powder (0.11 g/kg BW/d) experienced an increase in body weight gain from 0.127 kg/d in the control diet to 0.236 kg/d in the Spirulina platensis powder diet and a decrease in FCR from 13.20 to 7.35. The discrepancies in results may be attributed to factors such as the quantity and quality of feed provided as basal diets and Spirulina as supplement, as well as the animals’ previous health and nutrition status. Although Spirulina improved growth and feed efficiency, it did not significantly affect body condition score (BCS) or body dimensions (body length, heart girth, and wither height). However, it is important to note that BCS is a subjective measurement and may exhibit variability between assessments.

Spirulina’s potential growth-promoting effects may be related to its rich nutrient profile (Table 2), which includes a substantial protein content (53% to 75%), essential fatty acids, vitamins, and minerals that can supplement common diets [12,27,28,29,31,36]. In addition to its nutritional value, Spirulina’s interaction with gut microbiota in the rumen stimulates extracellular enzyme secretion [54], which enhances rumen feed absorption and promotes gut microbiota colonisation, ultimately leading to improved FCR.

### The benefits of Spirulina for rumen function

Meta-analysis results (Table 4), have revealed no significant impact of Spirulina supplementation on pH, acetate, butyrate, isobutyrate, propionate, or total volatile fatty acids (VFA) based on cumulative effect size. These findings are consistent with most studies, including Fomichev et al [26], who observed no significant effects of Spirulina on sheep rumen pH and VFA. However, Rabee et al [43], reported that Spirulina supplementation decreased VFA, acetate, and butyrate, with a concomitant increase in propionate and isobutyrate but no change in pH. Additionally, Plascencia et al [55] found that low-level Spirulina supplementation (up to 1% of diet) did not appreciably affect nutrient digestion, ruminal fermentation, or VFA in feedlot cattle fed a high-energy diet.

These studies generally indicate a minimal effect of Spirulina on rumen fermentation parameters in animals fed medium to high-quality diets with low level of Spirulina supplementation. Recent studies have shown that Spirulina can modulate rumen fermentation. Wang et al [12] found that Spirulina supplementation (3% of the diet) in a high-fat diet improved rumen development and fermentation in lambs, effectively improving rumen microbial health. This supplementation reduced daily feed intake while increasing acetate, propionate, butyrate, and total VFA production. These findings are consistent with those of Meteab et al [56], who observed that Spirulina supplementation (2 g/kg feed) enhanced the feed quality of Panicum maximum and alfalfa straw, optimising degradability and rumen fermentation parameters.

Additionally, Christodoulou et al [57] reported that high-level Spirulina supplementation (0.28 g/kg BW/day) in ewes slightly modulated rumen microbe composition with an increase in cellulolytic bacteria such as *Eubacterium ruminantium*, *Fibrobacter succinogenes*, and *Ruminococcus albus*. These bacteria may potentially improve the ability to degrade high-fibre diets in the rumen, leading to increased VFA production. Panjaitan et al [58] reported positive effects on rumen digestion in steers fed low-quality forage-based diets. Spirulina supplementation in these cases increased the concentration of branched-chain fatty acids, microbial protein production, feed intake, and decreased digesta retention time in the rumen. Costa et al [9] concluded that Spirulina can be considered a supplemental option to improve rumen function for grazing ruminants.

### The benefits of Spirulina on blood antioxidant status

The benefits of Spirulina on blood plasma or serum antioxidant parameters in small ruminants supplemented with various levels of Spirulina (cumulative effect size with 95% CI), are shown in Table 5. The results revealed significant effects of Spirulina supplementation on catalase (CAT) (1.26±0.20), glutathione peroxidase (GPx) (1.69±0.29), glutathione (GSH) (1.35±0.21), superoxide dismutase (SOD) (3.05±0.46), vitamin A (vit-A) (3.08±1.21), and malondialdehyde (MDA) (−0.51±0.15), all with large effect sizes except for MDA, which showed a medium effect size. Levels of CAT, GPx, GSH, SOD, and vit-A in serum or plasma increased significantly compared to the control group, while MDA levels decreased after Spirulina supplementation.

These findings suggest that Spirulina supplementation can enhance the antioxidant status of small ruminants. Enhanced antioxidant enzyme activity and reduced MDA levels indicate a reduction in oxidative stress, which may have positive implications for animal health and productivity. El-Sabagh et al [40] reported a significant increase in GSH and Vitamin A, along with a decrease in MDA, which were associated with a significant increase in ADG of fattening lambs fed common basal diets supplemented with Spirulina. Spirulina supplementation seems to effectively improve the antioxidant status of small ruminants, potentially benefiting their health and productivity.

### The benefits of Spirulina on blood biochemical profiles

The benefits of Spirulina on several blood biochemical parameters in small ruminants supplemented with various levels of Spirulina (cumulative effect size with 95% CI) are shown in Table 6. The results revealed significant effects of Spirulina supplementation on several blood biochemical parameters in small ruminants. Notably, levels of haemoglobin (2.11±0.53), total protein (0.79±0.20), urea (1.44±0.49), and white blood cells (0.94±0.27) increased, suggesting improvements in oxygen-carrying capacity, protein synthesis, kidney function, and immune response, respectively. All these parameters showed large effect sizes. Conversely, levels of alanine transaminase (ALT; −2.67±0.51), aspartate transaminase (AST; −3.04±0.46), cholesterol (−0.58±0.27), and low-density lipoprotein (LDL −0.76±0.27) decreased, indicating an improved lipid profile. ALT and AST showed large effect sizes, while cholesterol and LDL showed medium effect sizes. Healthy animals typically exhibit low levels of ALT and AST in their blood. Elevated ALT and AST often signal liver damage [59]. The meta-analysis demonstrated that small ruminants supplemented with Spirulina had lower ALT and AST levels than the control group. While the exact mechanism underlying these changes in ALT and AST concentrations remains unclear, Spirulina supplementation, as reported by El-Sabagh et al [40], was associated with an increase in feed intake, liveweight gain in fattening lambs, and a significant elevation in blood haemoglobin concentration, globulin concentration, white blood cells, and blood urea concentration. These findings suggest that Spirulina supplementation positively influenced blood biochemical profiles, likely due to its high protein and phytochemicals content, which may contribute to the overall health and productivity of small ruminants.

### The benefits of Spirulina on specific fatty acid profiles

Spirulina is known to be rich in fatty acids, and feeding it as a supplement to animals may modify the fatty acid composition of meat. The benefits of Spirulina on specific fatty acid profiles in small ruminants supplemented with various levels of Spirulina (cumulative effect size with 95% CI) are shown in Table 7. Spirulina supplementation altered the fatty acid profile of the *longissimus dorsi* muscle, increasing the proportion of eicosapentaenoic acid (EPA; 20:5ω3, an unsaturated fatty acid) and decreasing the proportion of palmitic acid (16:0, a saturated fatty acid). No significant effects were observed on other fatty acid profiles.

Overall, this study did not reveal significant effects of Spirulina supplementation on the majority of body fatty acid composition. However, Kashani et al [36] reported that polyunsaturated fatty acids such as EPA, which constitutes 1.95% of Spirulina’s total fatty acid content, were increased in subcutaneous adipose, muscle, and liver tissues of lambs grazing ryegrass pasture when Spirulina was supplemented at medium (0.33 g/kg BW/d) and high (0.66 g/kg BW/d) compared to the control group. Interestingly, while Spirulina was high in saturated palmitic acid (24.8% of total fatty acid), a main contributor to blood plasma cholesterol and LDL, its concentration did not increase in all examined tissues and was even lower than the control in heart and muscle tissue. These findings indicate that the effect of Spirulina on fatty acid profiles is variable and contentious.

### The benefits of Spirulina for meat and eating quality

Meta-analysis results (Table 8) revealed significant effects of Spirulina supplementation on key meat quality attributes in small ruminants (cumulative effect size with 95% CI). Meat pH increased (0.67±0.31) with a medium effect size compared to controls, while fat melting point (FMP) decreased (−1.94± 0.75) with a large effect size. Intramuscular fat percentage and tenderness also decreased significantly (−2.85±0.93 and −1.72±0.57, respectively) with large effect sizes. Although the meta-analysis showed an increase in meat pH with Spirulina supplementation, this change is biologically small and may not significantly impact actual meat quality. Al-Yahyaey et al [45] suggested that an increase in *longissimus dorsi* pH from (5.19±0.09) in the control diet to (5.84±0.17) in the treatment (T1) diet had no effect on other meat quality parameters. This aligns with Alghonaim et al [60], who reported no effect of Spirulina supplementation on meat pH but observed increased body wall fat, back fat thickness, and meat fat content, along with decreased muscle shear force. Consistent with this, Holman et al [29] demonstrated that elevated Spirulina supplementation in sheep was associated with a decreased FMP.

Interestingly, the meta-analysis results showed that the decrease in FMP was not directly proportional to IMF percentage. These findings suggest that Spirulina supplementation has mixed results on meat quality parameters. However, the meat industry prioritises increasing omega-3 and polyunsaturated fatty acids while concurrently reducing FMP to enhance meat tenderness [61]. Typically, lamb exhibits a fat melting point within the range of 38°C–55°C [62]. However, no significant effects were observed on sensory attributes such as appearance, aroma, juiciness, and taste.

### Bias analysis

**Figure 2:**
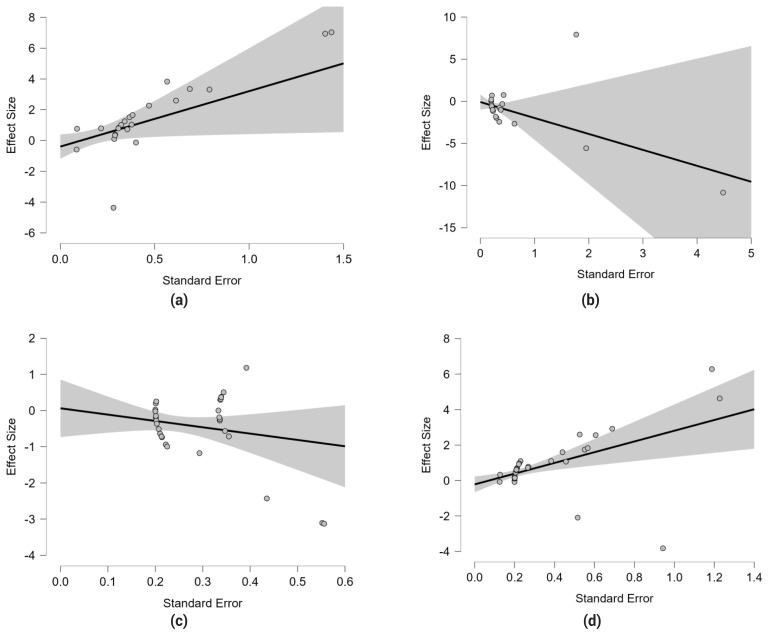
PET regression plot. (a) ADG, (b) cholesterol, (c) MDA, and (d) total protein. PET, precision-effect test; ADG, average daily gain; MDA, malondialdehyde.

A publication bias analysis was conducted for the four variables with the largest number of studies: ADG, cholesterol, MDA, and total protein. Neither PET-PEESE analysis nor sensitivity analysis using Rosenthal’s fail-safe N (Table 9) detected evidence of publication bias (p>0.05). Consequently, it was concluded that the analysed effect sizes were not subject to publication bias. These findings were visually corroborated by PET regression plots (Figure 2A–2D).

## CONCLUSION

The meta-analysis revealed that Spirulina supplementation improved feed intake, FCR, and ADG, along with meat quality, which was characterized by increased EPA and decreased shear force and FMP. These findings suggest that Spirulina has the potential to be a valuable dietary supplement, enhancing the economic viability of small ruminant production systems. However, these findings may be limited by the inclusion criteria and heterogeneity of the included studies. Future studies should investigate the optimal Spirulina dosage for various basal diets, its interactions with other dietary components, and its long-term effects on animal health and productivity. The nutritional composition of Spirulina suggests a strong potential for improving the performance of small ruminants fed low-quality diets, a common practice globally. Additionally, exploring the underlying mechanisms of Spirulina’s beneficial effects would provide further insights into its potential applications for sustainable livestock production.

## Figures and Tables

**Table 1:** Description of studies included in the meta-analysis of Spirulina supplementation on small ruminant performance and product attributes

References	Duration (days)	Dose (g/kg BW/day)	Species	Country
Fomichev et al [26]	14	0.02–0.03	Sheep	Russia
Al-Yahyae et al [27]	70	0.08–0.25	Goat	Oman
Malau-Aduli and Holman [28]	42	0.50–1.00	Sheep	Australia
Holman et al [29]	63	0.25–1.00	Sheep	Australia
Ghosh et al [30]	105	0.04–0.18	Goat	Bangladesh
Liang et al [31]	74	0.30–0.90	Sheep	China
Malau-Aduli and Kashani [32]	63	0.67–1.33	Sheep	Australia
Burezq and Khalil [33]	182	2.14	Sheep	Kuwait
Assar et al [15]	150	0.09–0.45	Sheep	Egypt
Hanafy [14]	42	0.10	Sheep	USA
Eldaim et al [34]	45–60	0.10	Sheep	Egypt
Khalifa et al [35]	90	0.01	Goat	Egypt
Mansour and Zeitoun [13]	168	0.23–0.46	Sheep	Egypt
Kashani et al [36]	63	0.17–0.67	Sheep	Australia
Sarvinda et al [37]	60	0.45–0.50	Sheep	Indonesia
Baliarti et al [38]	30	0.58	Sheep	Indonesia
Holman et al [39]	60	0.26–0.53	Sheep	Australia
El-Sabagh et al [40]	35	0.10	Sheep	Egypt
Wang et al [12]	74	0.90	Sheep	China
El-Deeb et al [41]	150	0.05	Goat	Egypt
Holman et al [42]	63	0.25–1.00	Sheep	Australia
Rabee et al [43]	42	0.13	Sheep	Egypt
Flakemore [44]	60	0.33–0.67	Sheep	Australia
Al-Yahyaey et al [45]	70	0.12–0.24	Goat	Oman

**Table 2:** Chemical composition and nutritional profile of Spirulina from various sources included in the meta-analysis

Nutrient composition in dry matter basis	Amount	Unit
Dry matter	78–96	%
Ash	6–12	%
Crude protein	53–75	%
Crude fibre	3–6	%
Crude lipid	7	%
Ether extract (EE)	1–5	%
Neutral detergent fibre (NDF)	2–38	%
Acid detergent fibre (ADF)	2–18	%
Metabolisable energy	11–17	MJ/kg
Fatty acid		
16:0 palmitic	24.8–44.9	%FA
18:0 stearic	1.7–6.3	%FA
20:0 arachidic	6.2	%FA
18:1 cis 9 (oleic)	9.8–16.6	%FA
18:1 trans 9 (elaidic)	2.8	%FA
16:1 palmitoleic	2.3–3.8	%FA
18:2 cis 9,12 (linoleic)	11.1–12.2	%FA
18:3 cis9,12,15 α-Linolenic	4.3	%FA
18:3 cis6,9,12 ϒ-Linolenic	24.4	%FA
20:4 cis5,8,11,14 (eicosatetraenoic)	13.9	%FA
Saturated fatty acids (SFA)	48.8–52.1	%FA
Monounsaturated fatty acids (MUFA)	5.1–10.4	%FA
Polyunsaturated fatty acids (PUFA)	37.5–46.0	%FA
Amino acids		
Phenylalanine	3.2	mg/kg
Valine	4.3	mg/kg
Threonine	4.7	mg/kg
Tryptophan	0.4	mg/kg
Methionine	1.0	mg/kg
Leucine	5.7	mg/kg
Isoleucine	3.9	mg/kg
Lysine	3.6	mg/kg
Histidine	5.1	mg/kg
Arginine	3.6	mg/kg
Minerals		
Calcium	2,070–2,700	mg/kg
Phosphorus	7,280–9,700	mg/kg
Zinc	1.9–17.6	mg/kg
Magnesium	3,200–4,860	mg/kg
Potassium	16,750–17,000	mg/kg
Iron	539–162	mg/kg
Manganese	10–24	mg/kg
Vitamins		
Riboflavin (B2)	55–81	mg/kg
Folic acid	8	mg/kg
Niacin (B3)	150–555	mg/kg
Pantothenic acid	179	mg/kg
Ascorbic acid	1,150	mg/kg

Data from Al-Yahyaey [27], Malau-Aduli and Holman [28], Liang et al [31], Wang et al [12], Holman et al [42], Kashani et al [36], Rahim et al [47], Raji et al [48], Raczyk et al [49], Ramírez-Rodrigues et al [50], Liestianty et al [51].

**Table 3:** Meta-analysis of growth performance and body conformation in small ruminants supplemented with various level of spirulina with 95% confidence intervals (CI)

Categories	Ns (n)	Xc	Xe	Standardized mean difference (continuous random-effects model)						

				Model results			Heterogeneity			
	
				Estimate	Standard error	p-value	Tau^2^	Q	Het. p-value	I^2^
ADG (g/d)	30	110.72	135.44	1.20	0.40	0.003	3.31	268.92	<0.001	91.08
DMI (kg/d)	6	1.07	1.10	−0.52	0.51	0.305	1.38	49.10	<0.001	89.82
FCR (g/kg)	11	0.01	0.01	−1.64	0.42	<0.001	1.53	65.49	<0.001	84.73
BCS (1–5)	10	2.31	2.33	0.19	0.20	0.354	0.24	23.14	0.006	61.11
BL (cm)	13	54.30	54.11	0.50	0.26	0.058	0.64	48.44	<0.001	75.23
HG (cm)	14	65.38	64.66	−0.17	0.32	0.602	1.16	97.46	<0.001	86.66
WH (cm)	13	53.98	52.51	−0.34	0.27	0.204	0.64	50.60	<0.001	76.28

Ns, number of studies; Xc, mean value of the control group; Xe, mean value of the treatment group; ADG, average daily gain; DMI, dry matter intake; FCR, feed conversion ratio; BCS, body condition score (0–5); BL, body length; HG, heart girth; WH, wither height.

**Table 4:** Meta-analysis of the effects of Spirulina supplementation on rumen fermentation parameters (pH, acetate, butyrate, isobutyrate, propionate, and total VFA’s) in small ruminants (95% CI)

Items	Unit	Ns	Xc	Xe	Standardized mean difference (continuous random-effects model)						

					Model results			Heterogeneity			
	
					Estimate	Standard error	p-value	Tau^2^	Q	Het. p-value	I^2^
Rumen profile											
pH	-	4	6.63	6.78	0.80	0.64	0.213	1.22	17.72	<0.001	83.07
Acetate	mmol/L	3	34.02	27.55	−0.80	0.53	0.132	0.54	7.27	0.026	72.50
Butyrate	mmol/L	3	5.03	4.36	−0.29	0.24	0.239	0.01	2.15	0.341	7.14
Isobutyrate	mmol/L	3	0.60	1.05	0.82	0.54	0.129	0.58	7.61	0.022	73.73
Propionate	mmol/L	3	12.62	16.71	0.13	0.86	0.877	1.81	18.05	<0.001	88.92
VFA	mmol/L	4	19.11	20.12	−0.9	1.12	0.407	4.58	49.08	<0.001	93.89

VFA, volatile fatty acid; CI, confidence interval; Ns, number of studies; Xc, Mean value of the control group; Xe, mean value of the treatment group.

**Table 5:** Meta-analysis of the effects of Spirulina on blood plasma or serum antioxidant parameters in small ruminants (95% CI)

Item	Ns	Xc	Xe	Unit	Standardized mean difference (continuous random-effects model)						

					Model results			Heterogeneity			
	
					Estimate	Standard error	p-value	Tau^2^	Q	Het. p-value	I^2^
CAT	16	445.52	496.89	U/mL	1.26	0.20	<0.001	0.27	25.41	0.045	40.97
GPx	16	84.50	101.07	U/mL	1.69	0.29	<0.001	0.81	42.74	<0.001	64.90
GSH	21	16.58	25.21	U/mL	1.35	0.21	<0.001	0.57	59.23	<0.001	66.23
MDA	33	7.88	4.78	U/mL	−0.51	0.15	<0.001	0.39	76.55	<0.001	58.19
SOD	26	70.74	88.10	U/mL	3.05	0.46	<0.001	4.66	281.92	<0.001	91.13
Vit-A	4	52.65	64.23	μg/dL	3.08	1.21	0.011	4.75	19.85	0.001	84.89

CI, confidence interval; Ns, number of studies; Xc, Mean value of the control group; Xe, mean value of the treatment group; CAT, catalase; GPx, glutathione peroxidase; GSH, glutathione; MDA, malonaldehyde; SOD, superoxide dismutase; Vit-A, vitamin A.

**Table 6:** Meta-analysis of the effects of Spirulina on blood biochemistry parameters in small ruminants (95% CI)

Item	Unit	Ns	Xc	Xe	Standardized mean difference (continuous random-effects model)						

					Model results			Heterogeneity			
	
					Estimate	Standard error	p-value	Tau^2^	Q	Het. p-value	I^2^
Total protein	g/dl	30	14.75	17.28	0.79	0.20	<0.001	0.85	119.91	<0.001	75.82
Albumin	g/dl	9	2.57	2.49	−0.37	0.61	0.543	2.87	61.74	<0.001	87.04
Globulin	g/dl	9	2.95	3.28	1.36	0.70	0.052	3.80	71.08	<0.001	88.75
ALT	IU/mL	22	37.44	24.19	−2.67	0.51	<0.001	5.00	264.03	<0.001	92.05
AST	IU/mL	26	68.82	51.97	−3.04	0.46	<0.001	4.68	292.15	<0.001	91.44
HDL	mg/dl	6	25.04	24.54	−0.85	0.47	0.073	0.96	18.32	0.003	72.70
LDL	mg/dl	6	22.22	15.73	−0.76	0.27	0.005	0.12	7.01	0.220	28.67
Cholesterol	mg/dl	27	65.21	61.11	−0.58	0.27	0.034	1.59	160.61	<0.001	83.81
Glucose	mg/dl	21	57.29	57.18	0.14	0.22	0.532	0.75	84.55	<0.001	76.35
Haemoglobin	g/L	9	122.29	131.98	2.11	0.53	<0.001	1.93	36.97	<0.001	78.36
TG	mg/dl	20	65.52	66.68	−0.43	0.35	0.218	2.25	313.43	<0.001	93.94
Urea	mg/dl	11	51.99	54.59	1.44	0.49	0.003	2.18	74.18	<0.001	86.52
WBC	10^3^/μL	10	9.24	10.95	0.94	0.27	<0.001	0.34	16.92	0.050	46.79

CI, confidence interval; Ns, number of studies; Xc, Mean value of the control group; Xe, mean value of the treatment group; ALT, alanine aminotransferase; AST, aspartate aminotransferase; HDL, high density lipoprotein; LDL, low density lipoprotein; TG, triacylglycerol; WBC, white blood cells.

**Table 7:** Meta-analysis of fatty acid composition in longissimus dorsi muscle of small ruminants supplemented with Spirulina (95% CI)

Item^1)^	Ns	Xc	Xe	Unit	Standardized mean difference (continuous random-effects model)						

					Model results			Heterogeneity			
	
					Estimate	Standard error	p-value	Tau^2^	Q	Het. p-value	I^2^
16:0	3	23.80	22.27	%FA	−4.42	1.37	0.001	4.95	19.39	<0.001	89.69
17:0	3	1.40	1.40	%FA	0.19	0.59	0.744	0.51	3.92	0.048	74.51
18:0	3	20.10	19.93	%FA	−0.08	0.24	0.747	0.00	0.69	0.710	0.00
18:1ω9	3	35.50	36.13	%FA	0.18	0.24	0.440	0.00	0.33	0.848	0.00
18:2ω6	3	4.50	3.87	%FA	−0.34	0.24	0.156	0.00	0.19	0.910	0.00
18:3ω3	3	2.00	1.90	%FA	−0.17	0.29	0.466	0.00	1.10	0.577	0.00
20:4ω6	3	0.70	0.63	%FA	−0.13	0.24	0.583	0.00	0.61	0.736	0.00
20:5ω3	3	0.10	0.40	%FA	0.81	0.28	0.004	0.06	2.62	0.270	23.65
22:5ω3	3	0.20	0.30	%FA	0.27	0.24	0.249	0.00	0.91	0.636	0.00
SFA	3	46.60	46.93	%FA	0.08	0.24	0.740	0.00	1.010	0.603	0.00
MUFA	3	44.80	45.40	%FA	0.17	0.24	0.486	0.00	0.721	0.697	0.00
PUFA	3	8.60	7.70	%FA	−0.27	0.24	0.253	0.00	0.562	0.755	0.00
ω-3	3	2.90	2.70	%FA	−0.21	0.32	0.505	0.14	3.59	0.116	44.25
ω-6	3	5.50	4.73	%FA	−0.31	0.24	0.186	0.00	0.128	0.938	0.00

1) 16:0, palmitic acid; 17:0, margaric acid; 18:0, stearic acid; 18:1ω9, oleic acid; 18:2ω6, linoleic acid; 18:3ω3, α-Linolenic acid; 20:4ω6, eicosatetraenoic acid; 20:5ω3, eicosapentaenoic acid; 22:5ω3, docosapentaenoic acid-3; ω-3, omega-3; ω-6, omega 6.

CI, confidence interval; Ns, number of studies; Xc, Mean value of the control group; Xe, mean value of the treatment group; SFA, saturated fatty acids; MUFA monounsaturated fatty acid; PUFA, polyunsaturated fatty acid.

**Table 8:** Meat quality, and eating quality of small ruminants supplemented with Spirulina

Item	Ns	Xc	Xe	Unit	Standardized mean difference (continuous random-effects model)						

					Model results			Heterogeneity			
	
					Estimate	Standard error	p-value	Tau^2^	Q	Het. p-value	I^2^
Meat quality											
FMP	11	44.15	43.27	ºC	−1.94	0.75	0.010	5.31	94.93	<0.001	89.47
IFP	5	2.80	2.10	%	−2.85	0.93	0.002	3.61	29.66	<0.001	86.51
pH	9	5.92	6.06		0.67	0.31	0.028	0.45	17.51	0.025	54.32
Eating quality											
Appearance	6	7.43	7.17	1–10	−0.97	0.91	0.287	4.41	46.07	<0.001	89.15
Aroma	6	7.27	6.81	1–10	−1.13	1.07	0.292	6.25	56.75	<0.001	91.19
Juiciness	6	7.39	6.99	1–10	−0.83	0.52	0.112	1.20	19.65	0.001	74.56
Taste	6	7.52	7.05	1–10	−1.03	1.13	0.362	7.02	60.47	<0.001	91.73
Tenderness	6	7.96	7.19	1–10	−1.72	0.57	0.003	1.41	19.47	0.002	74.32

Ns, number of studies; Xc, Mean value of the control group; Xe, mean value of the treatment group; FMP, fat melting point; IFP, intramuscular fat percentage.

**Table 9:** File drawer analysis^1)^

Item	Ns	Fail-safe N value	Significance of bias
ADG	30	994	Not significant
Cholesterol	27	842	Not significant
MDA	33	835	Not significant
Total protein	30	1,796	Not significant

1) Data from Rosenthal [25].
